# Supplementary material for: Transformer-Based HER2 Scoring in Breast Cancer: Comparative Performance of a Foundation and a Lightweight Model
Source: Diagnostics (Basel). 2025 Aug 23;15(17):2131. doi: 10.3390/diagnostics15172131 (PMC12428388; doi:10.3390/diagnostics15172131)
Supplement: Supplementary file 1 [file diagnostics-15-02131-s001.zip › diagnostics-3770796-supplementary.pdf]

**Supplementary Materials**

**Figure S1.** Training curves of two models.

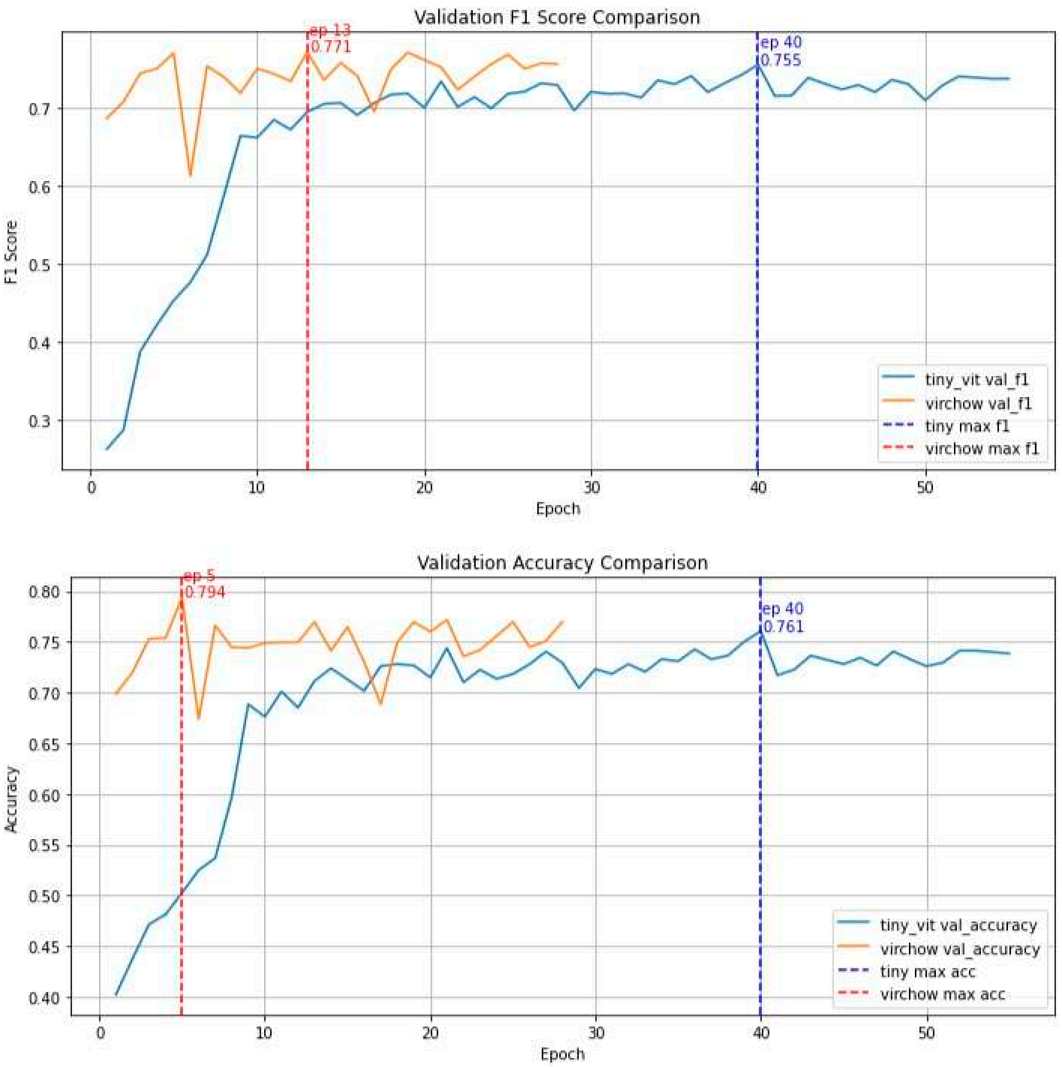

**Figure S2.** Patches misclassified by models. (A) Non-tumor as 1+, (B) 0 as 1+, (C) 1+ as 2+, (D) 2+ as 3+.

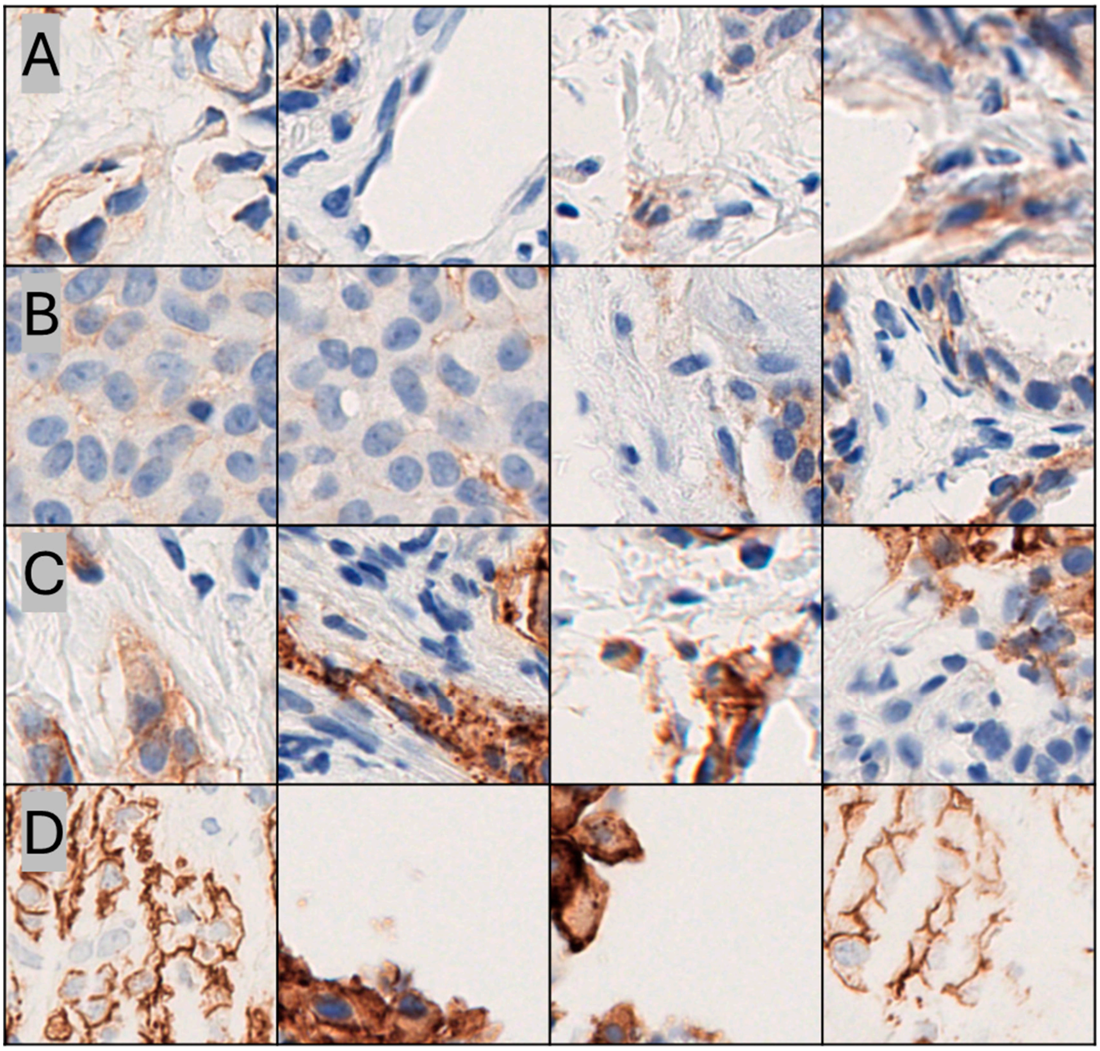

**Table S1.** Computational complexity, computational parameters and training hyperparameters of two models.

| | Virchow | TinyVit |
|---|---|---|
| Computational Parameters | | |
| Parameters | 632 | 21 |
| GFLOPs | 227.3 | 8.17 |
| Latency (ms) | 39.85 | 10.07 |
| Throughput (patches/s) | 34.91 | 784.76 |
| Peak GPU Memory (GB) | 5.58 | 0.78 |
| Training Hyperparameters | | |
| Image size | 224x224 | 224x224 |
| Batch size | 8 | 256 |
| Initial learning rate | 1.00E-04 | 1.00E-04 |
| Optimizer | Adam (lr=1e-4, betas=(0.9, 0.999), eps=1e-8, weight_decay=0) | Adam (lr=1e-4, betas=(0.9, 0.999), eps=1e-8, weight_decay=0) |
| Loss function | CrossEntropy | CrossEntropy |
| Scheduler | CosineAnnealingLR (T_max=20, eta_min=1e-5) | CosineAnnealingLR (T_max=20, eta_min=1e-5) |
| Early stopping patience | 15 | 15 |
| Data augmentations | HorizontalFlip, VerticalFlip, ColorJitter, Affine, GaussianBlur, MotionBlur | HorizontalFlip, VerticalFlip, ColorJitter, Affine, GaussianBlur, MotionBlur |
| Normalize | mean=[0.485, 0.456, 0.406], std=[0.229, 0.224, 0.225] | mean=[0.485, 0.456, 0.406], std=[0.229, 0.224, 0.225] |
| Mixed precision | TRUE | TRUE |
